# Metabolic and immune interaction between tuberculosis and diabetes mellitus: implications and opportunities for therapies

**DOI:** 10.1080/14656566.2025.2508904

**Published:** 2025-05-26

**Keywords:** TB, diabetes mellitus, insulin resistance, inflammation, host-directed therapy

## Abstract

**Introduction:**

Tuberculosis (TB) remains a major infectious threat to global health, while type 2 diabetes mellitus (diabetes) has reached epidemic proportions in many regions of the world. In low-and-middle income countries (LMIC) and among indigenous and minority communities in high-income settings (HIC), these diseases also increasingly overlap posing new clinical and therapeutic challenges.

**Areas covered:**

We searched PUB MED/CINAHL/Web of Science/Scopus, and Google Scholar up to 30 November 2024. While TB and diabetes are different conditions, their bidirectional relationship and immuno-metabolic parallels are underappreciated. Improved understanding of these mechanisms may pave the way for novel therapeutic strategies, for example, using antidiabetic medications as novel adjuvant host-directed therapies (HDT) in active TB. We review the epidemiology of TB, diabetes and their combined comorbidity, their immune and metabolic mechanisms and clinical relevance as well as potential opportunities for general and targeted therapeutic intervention.

**Expert opinion:**

The interaction between diabetes and tuberculosis is bidirectional with diabetes a predisposing factor to tuberculosis and vice-versa. Underlying this interaction are shared inflammatory immunometabolic mechanisms. It follows that treatments for diabetes and its complications may be beneficial in tuberculosis and that the treatment of both active and latent tuberculosis may improve glycaemic control. These interactions are amenable to investigation in experimental models, in human experimental medicine studies and in clinical trials.

## Introduction

Tuberculosis (TB) and type 2 diabetes mellitus (diabetes) are two major global health challenges, each causing substantial ill-health, disability and premature death. While different conditions, TB and diabetes are bidirectionally related. A growing body of evidence suggests complex interplay between TB and diabetes, with synergistic adverse impacts on incidence, progression and outcomes of each condition. Driving this bidirectional relationship are similar immune and metabolic derangements. Improved understanding of these interactions and their mechanistic correlates is important to develop needed novel interventions, especially novel therapeutic approaches in TB. Over the past five years, research has shed new light on this complex relationship between diabetes and TB.

### Epidemiology

Latent TB is considered to affect 2 billion people worldwide, with 10.1 million incident active TB cases and 1.3 million fatalities annually (WHO, 2025). Diabetes in turn affects 537 million people with 6.7 million deaths per year (IDF, 2021). These diseases increasingly overlap. It is estimated that 15% of patients with active TB globally have prevalent diabetes (Noubiap et al., 2019). This rises to nearly half of all active TB cases in such settings as the Marshall Islands (45.2%) (Nasa et al., 2014), Saudi Arabia (42.2%) (Al-Tawfiq and Saadeh, 2009), Mexico (54.4%) (Munoz-Torrico et al., 2017) and Pakistan (39.6%) (Aftab et al., 2017), (Noubiap et al., 2019). In contrast, the prevalence of TB among patients with diabetes ranges from 0.38% in Taiwan (Lin et al., 2015) to 14% in Pakistan (Amin S, 2011). In sub-Saharan Africa (SSA) where HIV is the main risk factor for TB, the pooled prevalence of diabetes in people with active TB is 8% (Noubiap et al., 2019).

This bidirectional relationship of TB and diabetes is also reflected in increased risks of incident TB. TB is a long-recognized complication of diabetes with the latter increasing the risk of new *Mycobacterium tuberculosis* (*M.tb*) infection as well as the risk of progressing to active TB from latent infection (Kamper-Jorgensen et al., 2015; Koesoemadinata et al., 2017). According to prospective observations, people with diabetes are about three-times [relative risk (RR) (95%CI): 3.6 (2.3 - 5.7)] more likely to develop active TB than their non-diabetic counterparts (Al-Rifai et al., 2017; Jeon and Murray, 2008). These prospective studies better establish the temporality of exposure and outcome, a crucial consideration given the bidirectionality of the TB-diabetes relationship.

Conversely, there is growing recognition that diabetes may also be a metabolic sequel of both latent and active infection with *M.tb*. A study using a UK nationwide cohort of adults enrolled in primary care (mean follow-up 4 years) reported adjusted incidence rate ratios (IRR) for new-onset diabetes of 5.7 following pulmonary TB (PTB) and 4.7 following extrapulmonary TB (EPTB) compared to the general population (Pearson et al., 2019). A US-wide study (median follow-up 3.2 years) found higher diabetes incidence in adults with reactive tuberculin skin testing (TST) and/or interferon-γ release assay (IGRA) than in non-reactive peers [adjusted HR (95%CI): 1.2 (1.2, 1.3)] (Magee et al., 2022). Of note, this study excluded patients with prior active TB and those diagnosed with diabetes within 2 years of TST and IGRA testing. In contrast, a prospective cohort in Oxford, England, did not find evidence that TB increases the risk of incident diabetes [RR (95%CI): 1.1 (0.8, 1.6)] (Young et al., 2012). Diabetes is a multifactorial disease, and it may be that infection with *M.tb* is a trigger for diabetes to manifest in individuals with predisposing factors. Notwithstanding, these epidemiological links are increasingly corroborated by mechanistic studies of TB in humans and animals showing increased insulin resistance, inflammation within adipose tissue (Agarwal et al., 2014; Ayyappan et al., 2018; Bisht et al., 2023), free fatty acid dysregulation and dysglycaemia (Magodoro et al., 2024), among other characteristics of diabetes.

### Clinical Manifestations

#### TB in Diabetes

Concurrent TB and diabetes can pose significant diagnostic challenges (Boadu et al., 2024b; Gil-Santana et al., 2016). For example, diabetes in people with TB has been associated with a considerably longer time (25 days versus 6 days in TB alone) to diagnosis and/or antituberculosis treatment initiation (Chen et al., 2014; Wang et al., 2017). Patients newly diagnosed with PTB in Beijing, China, had a median (IQR) 6 (3-31) days from time of onset of pulmonary symptoms to time of first contact with any formal health facility. Their counterparts with concurrent PTB and diabetes had 25 (5-61) days (Chen et al., 2014). Similarly, Wang et al., found that having hyperglycaemia *i.e*., either known diabetes, or newly identified diabetes or prediabetes at time of PTB diagnosis, was associated with greater likelihood [odds ratio (OR) (95%CI): 2.1 (1.5, 3.0)] of PTB diagnostic delay (defined as >28 days from symptom onset to first formal healthcare contact) compared to normoglycemic controls (Wang et al., 2017). However, there is also counter evidence suggesting that diabetes does not contribute to TB diagnostic delay, and that it may in fact associate with expedited anti-tuberculosis treatment (ATT) initiation (Xiao et al., 2021).

TB may exhibit greater clinical complexity due to diabetes-related complications such as end-organ damage or failure (Shetty et al., 2024). Commonly, TB in people with diabetes presents either as disseminated, or more infectious and severely cavitating forms of the disease with higher clinical severity scores (Boadu et al., 2024b; Gil-Santana et al., 2016; Huang et al., 2017; Shetty et al., 2024; van Crevel and Critchley, 2021; Zhao et al., 2024). Likewise, PTB patients with poorly controlled diabetes have a more severe clinical picture than peers with PTB and well controlled diabetes. Atypical radiographic features are not uncommon either, and include miliary TB, lower lobe infiltrates, pleural effusions, pulmonary nodules, and cavities involving the middle and lower lung zones (Boadu et al., 2024a; Rottenberg et al., 2017; Shetty et al., 2024; Stubbs et al., 2021; Zhao, 2024).

#### Diabetes in people with TB

Similarly, the presence of hyperglycaemia at the initial diagnosis of TB disease presents diagnostic and management challenges. This is particularly relevant in patients not previously known to have diabetes (Kubjane et al., 2020; Song et al., 2019). In non-diabetic individuals, hyperglycaemia typically normalizes during the first three months of ATT (Kubjane et al., 2020). Key gaps in knowledge remain about whether this transient hyperglycaemia warrants intervention and whether it affects the long-term risk of diabetes risk. Based on available evidence, guidelines (2010) recommend confirmatory diabetes testing three (3) months after initiation of TB treatment to avoid misclassification of transient hyperglycaemia as diabetes (Ottmani et al., 2010). However, the optimal retesting interval remains to be ascertained.

In contrast, hyperglycaemia may persist despite ATT in people with diabetes who are newly diagnosed with TB (Kubjane et al., 2020). Both TB and ATT can exacerbate existing hyperglycaemia complicating the pharmacological management of diabetes (Boadu et al., 2024a; Song et al., 2019). Furthermore, rising HbA1c levels during and after ATT relate to poor treatment response (Shetty et al., 2024). Importantly, HbA1c values are also influenced by co-existing factors such as anaemia (English et al., 2015) and antiretroviral medications like reverse transcriptase inhibitors (Dave et al., 2011; Kubjane et al., 2020), for example. These factors warrant consideration when interpreting the validity of the HbA1c result (Kubjane et al., 2020).

### Impact of diabetes comorbidity on TB treatment efficacy and outcomes

The interaction between TB and diabetes complicates the treatment of each condition with important repercussions for both clinical care and public health programmes (Table 1). For example, the high pill-burden in patients with comorbid diabetes and TB increases the likelihood of missed doses, incorrect drug intake and treatment interruptions (van Crevel and Critchley, 2021). These risks are pronounced with more difficult to control diabetes and/or MDR-TB. The recommended treatment period for the latter is 6 months, and it is not uncommon for it to be extended (Committee, 2014). In addition, people with diabetes have higher risks of developing serious adverse events and reactions to TB medication (Muñoz-Torrico et al., 2017; van Crevel and Critchley, 2021). Nephrotoxicity, hepatoxicity, visual acuity disturbances and hypothyroidism are examples of aggravated TB drugs adverse reactions in people with diabetes while isoniazid can aggravate diabetic neuropathy (Muñoz-Torrico et al., 2017; Syed Suleiman et al., 2012; van Crevel and Critchley, 2021).

Similarly, diabetes negatively impacts TB treatment outcomes. The risk of death during TB treatment is doubled with comorbid diabetes [OR (95%CI): 1.9 (1.6, 2.2)] (Huangfu et al., 2019). Likewise, the probability of treatment failure is higher in TB patients with diabetes compared to TB alone, as are rates of recurrence or relapse, extended treatment duration, delayed sputum culture conversion and the emergence of MDR-TB (Gautam et al., 2021; Huangfu et al., 2019; Khattak et al., 2024; van Crevel and Critchley, 2021; Zhao et al., 2024). The emergence of MDR-TB is particularly concerning. People with diabetes have comparatively poor control of *M.tb* infection (Zhao et al., 2024) with resultant increased mycobacterial proliferation, and therefore higher bacterial load (Abd El-Hamid El-Kady and Abdulrahman Turkistani, 2021; van Crevel and Critchley, 2021). Nosocomial acquisition of drug resistance TB is also relatively more common among people with diabetes (Rehman et al., 2023; van Crevel and Critchley, 2021). Overall, these outcomes are worse with poor versus optimal glycemic control among those with comorbid diabetes and TB (Mahishale et al., 2017; Zhao et al., 2024). Curiously, however, TB-related mortality risk [OR (95%CI): 0.6 (0.2, 1.5)] in people with diabetes may not be impacted by glycemic control according to a recent meta-analysis (Zhao et al., 2024).

#### *M.tb* infection impacts glucose metabolism

Adipocytes and adipose tissue may be the mechanistic bridge between *M.tb* infection and deranged glucose metabolism. *M.tb* preferentially infect cells of the myeloid lineage, like macrophages, as these are the first innate immune cells to encounter the bacterium upon infection (Chandra et al., 2022; Cliff et al., 2015). *M.tb* can also infect several non-traditional immune cell types, including adipocytes where they establish latency (Niazi and Kalra, 2012). Aerosolized *M.tb* initially infects the lungs and disseminates to adipose tissue where it remains latent, In the event of immune system compromise, such as during HIV infection or with diabetes, bacilli could reactivate and disseminate back to the lungs (and other sites). Adipose tissue throughout the body is susceptible and may, therefore, constitute a vast reservoir where *M.tb* can persist.

Adipose tissue with its triglyceride content is a nutritionally rich niche for the persistence of *M.tb*. It is also an endocrine organ contributing to metabolic and energy homeostasis. *M.tb* infection and persistence may have a dynamic effect on this physiology, setting off a cascade within the adipose tissue microenvironment of immune cell infiltration, activation and cytokine release, culminating in disruption of glucose, insulin, and lipid regulation (Das et al., 2024). *M.tb*-related metabolic disruptions are thought to mirror the inflammatory changes observed in adipose tissue in obesity-related insulin resistance, which often presages the onset of diabetes. Indeed, increased insulin resistance with hyperglycaemia has been demonstrated in both latent and active TB infection.

**Figure 1:**
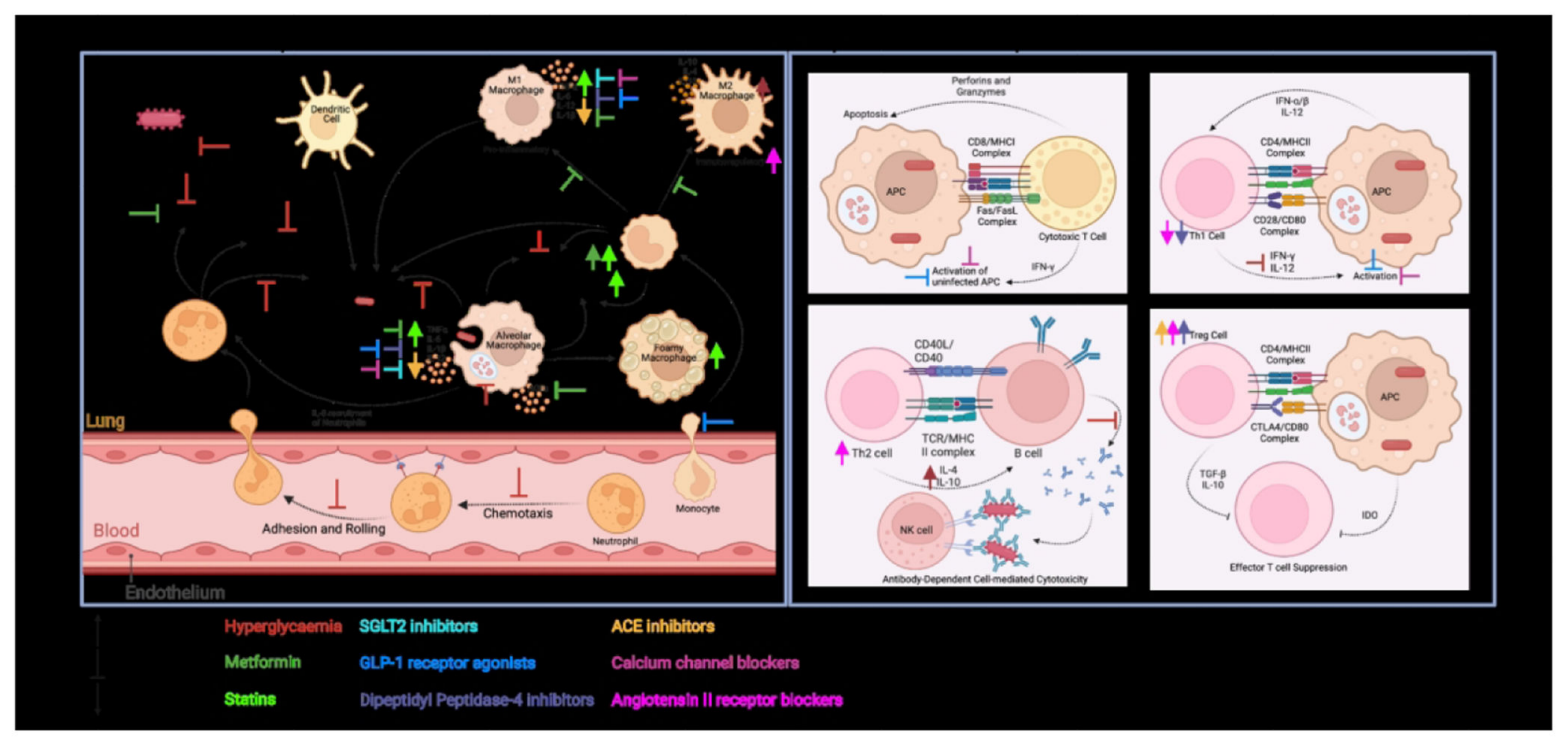
An overview of the Innate and Adaptive Immune Responses to M.tb infection and the effect of diabetes therapies thereon. Hyperglycaemia, a hallmark of type II diabetes, inhibits protective innate immune responses which can be improved with successful diabetes treatment. Diabetes therapies typically inhibit pro-inflammatory immune responses, favouring the upregulation of immunomodulatory responses. M.tb: Mycobacterium tuberculosis; IL: Interleukin; TNF*α*: Tumor necrosis factor alpha; AGEs: advanced glycation end-products; APC: antigen presenting cell; MHCI: major histocompatibility complex class 1; MHCII: major histocompatibility complex class 2; IFN: interferon; TCR: T cell receptor; NK: natural killer; IDO: Indoleamine 2,3-dioxygenase; TGF: transforming growth factor; Th1: helper T cell subset type 1; Th2: helper T cell subset type 2; Treg: regulatory T cell subset. Created in BioRender. Kotze, L. (2024) https://BioRender.com/l93g049.

Although diabetes is primarily a metabolic disorder, its accompanying host immune changes are often deleterious (Figure 1). Diabetes is associated with systemic inflammation, oxidative stress and aberrant cytokine production, among other alterations. These wide-ranging immune effector changes (Figure 1) associate with increased expression of genes associated with innate inflammatory responses, on one hand, and a decrease in those associated with adaptive immunity, on the other. For instance, decreased type I interferon responses in patients with TB and diabetes comorbid conditions, indicating an unexpected separation of the TB transcriptome phenotype, where increased type I responses are detrimental to the host (Eckold et al., 2021). This imbalance is also present in individuals with intermittent hyperglycaemia and TB comorbidity, demonstrating altered immune responses even under acute hyperglycaemic conditions, thus leading to a less effective immune response against TB (Eckold et al., 2021). Here, we discuss how various diabetes therapies further alter the host immune response to *M.tb* infection (interactions summarized in Figure 1).

### Pharmacological considerations

#### (1) Metformin

Metformin is a widely used oral anti-diabetic drug which, in the last decade, has gained attention as a potential adjuvant host-directed therapy (HDT) in TB (Chung et al., 2024; Salindri et al., 2024; Wang et al., 2022). Recent meta-analyses (Meregildo-Rodriguez et al., 2022a; Yu et al., 2019; Zhang and He, 2020), including only retrospective cohort studies, demonstrate decreased risk of incident active TB with the use of metformin versus none in people with diabetes. Further reports suggest metformin may reduce the risk of incident active TB in people with diabetes to levels seen in persons without diabetes (Pan et al., 2020a). The impact of metformin appears to be dose-dependent, with higher doses associating with greater risk reduction (Heo et al., 2021).

Noteworthy is that available studies are almost exclusively observational. (Padmapriydarsini et al., 2022) randomized 306 adults with newly diagnosed smear positive drug sensitive pulmonary TB to receive standard ATT or standard ATT with additional metformin during the first 8 weeks. People with diabetes were excluded. Participants in the metformin arm had a faster resolution of cavities on chest radiography, *i.e*., amelioration of lung pathology, and decreased levels of pro-inflammatory cytokines in plasma at 8 weeks of treatment. However, sputum culture conversion rates were similar across study arms. Although the evidence of metformin’s potential as an adjunctive therapy to antituberculosis treatment is growing, it is not unequivocal. In murine TB, for example, metformin use in both diabetic and non-diabetic mice has been associated with augmentation of bacillary load and lung immunopathology (Sathkumara et al., 2020).

Metformin’s effects on TB may not be related to glycemic control as they are not observed with other anti-diabetic medications (Fu et al., 2021). Metformin is thought to be an immunomodulator acting via immune cells, including autophagy, and various circulating immune mediators. Autophagy, an intracellular self-digestion process, is critical to the elimination of intracellular pathogens and plays an important role in defence against *M.tb* (Gutierrez et al., 2004). It is regulated by the mammalian target of rapamycin (mTOR) complex and adenosine monophosphate-activated protein kinase (AMPK), which activate the pathway. *In vitro* studies show that metformin activates AMPK (Singhal et al., 2014), resulting in increased autophagy and subsequently reduced intracellular *M.tb* growth; as well as inhibition of LPS-induced chemokine expression (Ye et al., 2018).

Another mechanism by which metformin is postulated to exert its effects is inhibition of mycobacterial growth by increasing macrophage viability and activation (Naicker et al., 2023), the production of the antimicrobial peptide β-defensin (Rodriguez-Carlos et al., 2020) and a direct effect on *M.tb* (Naicker et al., 2023). Metformin use in TB is associated with comparatively low levels of TNF-α, IFN-γ and IL-1β (pro-inflammatory cytokines) (Arai et al., 2010; Gonzalez-Muniz et al., 2024; Naicker et al., 2023; Padmapriydarsini et al., 2022; Roca et al., 2022; Ye et al., 2018) (Kumar et al., 2018; Li et al., 2017), advanced glycation end products (AGE) and soluble receptor for AGE (sRAGE) (Kumar et al., 2019). Moreover, metformin stimulates the differentiation of T-cells into both regulatory (Tregs) and CD8+ memory T cells, shifting the balance away from pro-inflammation. Metformin increases the mitochondrial mass, and oxidative phosphorylation and fatty acid oxidation capacity of CD8+ T cells. This metabolic reprogramming in turn enhances the ability of these cells to contain *M.tb* (Bohme et al., 2020).

More recently, steroid hormone synthesis has been highlighted as a possible effector of metformin’s antituberculosis actions (Gonzalez-Muniz et al., 2024). Cortisol reduces innate immune responses, while dehydroepiandrosterone (DHEA) is pro-inflammatory. In individuals with both TB and diabetes, these hormones become dysregulated, resulting in an increased cortisol/DHEA ratio. This immuno-endocrine imbalance is thought to impede an effective immune response to *M.tb*. (Gonzalez-Muniz et al., 2024) have examined *ex vivo* the impact of metformin on cortisol and DHEA synthesis in adrenal cells and how these hormones affect the expression of proinflammatory cytokines and antimicrobial peptides (AMP) in *M.tb*-infected macrophages. Metformin enhances DHEA synthesis while maintaining cortisol balance in adrenal cells. In turn, the mycobacterial load was reduced in infected macrophages by the increased production of proinflammatory cytokines (TNF-α, IL-12, IL-1β), the antimicrobial nitric oxide synthase, and AMP (CAMP, DEFB4, DEFB103) (Gonzalez-Muniz et al., 2024).

#### (2) Other antidiabetic agents

Metformin’s effects on TB appears to be independent of glycemic control as similar effects are not seen with other anti-diabetic medications (Fu et al., 2021). The recent meta-analysis by Meregildo-Rodriguez et al., did not find association between TB risk and the use of other common anti-diabetic drugs like sulfonylureas, meglitinides, thiazolidinediones and alpha-glucosidase inhibitors (Meregildo-Rodriguez et al., 2022a). This further underscores the importance of immunomodulation as a strategy for adjuvant HDT (Sun et al., 2012). Glucagon-like peptide (GLP)-1 receptor agonists stimulate insulin secretion in response to hyperglycaemia. Although the anti-inflammatory effects of these agents have been observed - including decreased C-reactive protein and IL-6 (Prasad-Reddy and Isaacs, 2015), inhibition of macrophage activation and diminished macrophage infiltration into tissues (Nesto, 2004) - their antitubercular benefits, if any, remain to be established.

Similarly, the immunomodulatory properties of dipeptidyl peptidase (DPP)-4 inhibitors are increasingly recognized and include altered T-helper cell responses, decreased pro-inflammatory Th1 and Th17 cells, and increased anti-inflammatory Tregs (Gallwitz, 2019; Kim et al., 2014). The net effect of these appears to be a reduction of pro-inflammatory cytokines IL-6, TNF-α, and MCP-1, which are associated with insulin resistance and chronic inflammation in type 2 diabetes (Agrawal and Kant, 2014). However, DPP-4 inhibitors have not been shown to impact the risk of developing TB (Wang et al., 2023).

SGLT2 inhibitors exhibit anti-inflammatory properties by decreasing the levels of pro-inflammatory cytokines like IL-6 and TNF-α. The effects have been studied in the context of cardiovascular benefit; this has been seen through mitigating systemic inflammation and enhancing endothelial function, particularly in patients with diabetes (Ahlstrom and Lamberg-Allardt, 1999; Esaki et al., 1998; Kurosaki and Ogasawara, 2013). Their impact, if any, on TB infection risk in people with diabetes is unknown.

#### (3) Statins

Like metformin, statins are widely used drugs which inhibit 3-hydroxy-3-methyl glutaryl (HMG)-CoA reductase, and thereby cholesterol biosynthesis (Istvan and Deisenhofer, 2001). Guidelines recommend statins for primary and secondary prevention of cardiovascular events in patients with diabetes (Marx et al., 2023). More recently, statins have been associated with beneficial effects in various infectious diseases (Ray et al., 2010; Vuorio and Kovanen, 2020), including TB. Three recent meta-analyses (Duan et al., 2020; Li et al., 2020; Meregildo-Rodriguez et al., 2022b) of retrospective cohorts, including than 2 million patients, found that the use of statins reduces the risk of incident active TB in people with (RR (95%CI): 0.78 (0.63, 0.95)) and without (RR (95%CI): 0.60 (0.50, 0.71)) diabetes (Duan et al., 2020).

Several mechanisms are thought to mediate these effects of statins. Improved phagosome maturation in murine macrophages with statins has been demonstrated (Parihar et al., 2014), as has decreased intracellular viability of *M.tb* in the presence of statins (Bruiners et al., 2020; Guerra-De-Blas et al., 2019; Lobato et al., 2014; Parihar et al., 2014). Some of these effects are dose-dependent. Statins modulate lymphocyte, including T helper 1 (T_H_1) and T_H_2 cells, and macrophage responses including their capacity to produce chemokines and cytokines (Guerra-De-Blas et al., 2019; Matsumoto et al., 2004; Montero-Vega et al., 2024), and autophagy (Guerra-De-Blas et al., 2019; Parihar et al., 2014).

*M.tb* uses cholesterol in the host macrophage membrane to bind and enter the macrophage (Gatfield and Pieters, 2000). It also accumulates host cholesterol in its own cell wall, thereby decreasing its permeability torifampicin. A reduction in cholesterol by statins may impair the entry of *M.tb* inside macrophages but improve the entry of rifampicin (Brzostek et al., 2009). Decreasing cholesterol levels also impacts the AMPK-mTORC1-TFEB axis leading to increased autophagy (Bruiners et al., 2020). Contradicting the latter, however, is the fact TB risk reduction has not been demonstrated with non-statin lipid lowering drugs (Pan et al., 2020b). Further, population-based studies suggest lower cholesterol levels may be associated with a higher risk of incident active TB (Jo et al., 2021). Thus, statin-mediated effects are likely driven by cholesterol-independent effects as well as the hydrophilic or lipophilic nature of these drugs (Bruiners et al., 2020; Brzostek et al., 2009; Davuluri et al., 2023; Dutta et al., 2016; Dutta et al., 2020; Gatfield and Pieters, 2000; Guerra-De-Blas et al., 2019; Lobato et al., 2014; Matsumoto et al., 2004; Montero-Vega et al., 2024; Parihar et al., 2014).

**Figure 2:**
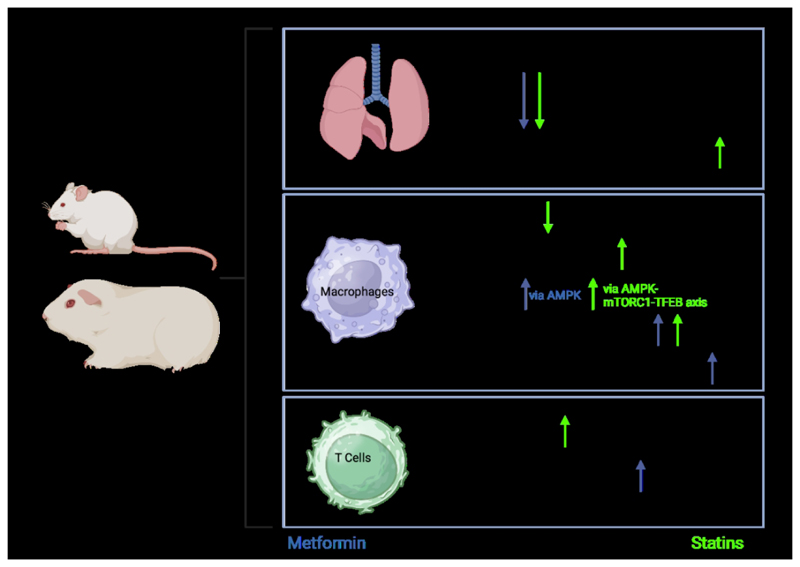
An overview of the influence of metformin and statins on the systemic and immune effects of M.tb infection in animal models, primarily those in mice and Guinea pigs. AMPK: adenosine monophosphate-activated protein kinase; mTORC1: mammalian target of rapamycin complex 1; TFEB: transcription factor EB; Th2: helper T cell subset type 2. Created in BioRender. Kotze, L. (2024) https://BioRender.com/l93g049.

Studies assessing the effect of statins on TB treatment outcomes are less frequent and have varied results. In mice and guinea pigs (Figure 2), statin use was associated with reduced mycobacterial burden in the lungs. This was observed in animals dosed with statins before TB infection and where statins were added as adjunct to TB treatment (Davuluri et al., 2023; Dutta et al., 2016; Dutta et al., 2020; Parihar et al., 2014). One retrospective cohort study including patients with comorbid TB and cardiovascular disease showed a lower overall mortality but no apparent improvement in infection-related mortality with statin use (Chidambaram et al., 2021). A study assessing patients with TB and diabetes found a beneficial effect of statins on TB treatment outcomes independent of glucose regulation (Meng et al., 2024). Statin use was also associated with a lower incidence of drug-induced liver injuries during TB treatment (Huang et al., 2024).

Two RCT have tested the effect of standard ATT versus standard ATT plus adjunctive statins on sputum culture conversion at 8 weeks in humans with PTB. (Cross et al., 2023) included 137 participants from the Philippines, Vietnam and Uganda, and found rosuvastatin had no effect on time to culture conversion. In contrast, (Adewole et al., 2023) found higher frequency (97% vs. 85%; p=0.02) of sputum culture conversion at 8 weeks and a greater reduction in chest radiograph severity scores with atorvastatin among patients with PTB in Nigeria. Both trials found statin use was not associated with an overall increase in adverse events.

#### (4) Antihypertensives and other drug classes

Various classes of antihypertensive drugs have immunomodulatory actions. These include angiotensin-converting enzyme inhibitors (ACEI), angiotensin II receptor blockers (ARB), and calcium channel blockers (CCB). Variously these actions include dampening of the inflammatory response via lowering pro-inflammatory cytokines like TNF-α, IL-6, and IL-1β, and/or enhancing anti-inflammatory cell responses (Ambrosioni et al., 2001; Brown and Ellis, 1969; Hagiwara et al., 2009; Tuite, 1992); (Benicky et al., 2009; Crowley, 2014; Gurlek et al., 2001; Kasal and Schiffrin, 2012; Sierra and de la Sierra, 2005; Silveira et al., 2013). The latter entails, for example, moving away from a pro-inflammatory Th1 profile and towards a more anti-inflammatory Th2 and T-regulatory cell profile (Arbues et al., 2020; Cardona and Cardona, 2019; Chatterjee et al., 2021; Cooper and Khader, 2008; Pahari et al., 2018; Shakya et al., 2012; Shi et al., 2019), or inducing macrophage polarisation towards the anti-inflammatory M2 phenotype (Carson et al., 2001; Dhande et al., 2015; Schmieder, 2005). There are additional actions like mitigating oxidative stress by ACEI via inhibition of nicotinamide adenine dinucleotide phosphate (NADPH) oxidase activity. This in turn potentially limits tissue damage (Ambrosioni et al., 2001; Brown and Ellis, 1969; Hagiwara et al., 2009; Tuite, 1992).

Whether these immunomodulatory effects can modulate TB risk, and/or improve treatment is a matter of ongoing investigation. One nested case–control analysis using a Taiwanese nationally-representative longitudinally followed cohort (772,000 person-years) found current use of ACEI to be associated with a decreased risk of incident active TB, particularly with chronic use (>90 days), showing a duration-response effect (Wu et al., 2016). Chronic ACEI use was associated with a 26% decrease in TB risk compared to non-users (RR (95%CI): 0.74 (0.66, 0.83)). The decrease in TB risk was also consistent across all age, sex and cardiovascular comorbidity patient subgroups. Meregildo-Rodriguez et al., have recently shown in a meta-analysis including 4 million participants that CCB reduce the risk of developing active TB by 29% (RR (95%CI): 0.71 (0.67, 0.75)) with and without diabetes mellitus (Meregildo-Rodriguez et al., 2024). These protective effects of CCB were independent of the class of CCB used.

### Drug interactions and pharmacokinetic implications

Diabetes may impact antitubercular drug pharmacokinetics. Altered body composition in diabetes impacts the distribution of lipophilic drugs like rifampicin and pyrazinamide, potentially leading to subtherapeutic concentrations (McIlleron et al., 2006; Weiner et al., 2004). Hepatic metabolism of isoniazid, rifampicin, and pyrazinamide may be impaired by non-alcoholic fatty liver disease, common in diabetes, increasing risks of toxicity or reduced efficacy (Ruslami et al., 2010). Chronic kidney disease, frequently observed with diabetes, can also reduce clearance of drugs like ethambutol and streptomycin, heightening toxicity risks, notably ethambutol-induced optic neuropathy (Cevik et al., 2024; Ruslami et al., 2010; Sekaggya-Wiltshire and Dooley, 2019).

Concomitant medications for diabetes management also impact tuberculosis treatment. Rifampicin increases metformin exposure without altering its glucose-lowering effects, while metformin reduces exposure to and accelerates clearance of rifampicin, isoniazid, and pyrazinamide (Padmapriydarsini et al., 2022; Te Brake et al., 2019). *In vitro*, metformin enhances rifampicin and isoniazid activity but hinders ethambutol’s (Trivedi and Chaturvedi, 2023). Rifampicin also reduces simvastatin plasma concentrations (Dutta et al., 2016; Skerry et al., 2014; Kyrklund et al., 2000), necessitating alternative statins like pravastatin or rosuvastatin during tuberculosis treatment. Clinicians should be aware of potential myopathy risk with combined isoniazid and simvastatin therapy (Alffenaar et al., 2016). Therefore, careful consideration of body composition, organ function, and drug-drug interactions is crucial when treating tuberculosis in patients with diabetes. Dose adjustments or therapeutic drug monitoring may be necessary to optimize outcomes and minimize adverse events. Lastly, corticosteroids are regularly prescribed as adjunctive therapy in the treatment of TB, especially in meningeal or pericardial TB or for the treatment or prevention of paradoxical TB immune reconstitution inflammatory syndrome. Their use can lead to hyperglycaemia or dysregulation of existing DM, also in patients with TB (Schutz et al., 2018).

### Prevention of TB in people with diabetes

The increased risk of TB and its associated morbidity and mortality underscores the need for targeted interventions to prevent TB. Treatment of TB infection, also known as TB preventive treatment (TPT), is globally recommended for populations at increased risk of TB, such as people living with HIV and individuals who are household contacts of persons with active TB. However, the global recommendation on TPT in people with diabetes does not exist currently unless they belong to risk groups that are eligible for TPT (WHO, 2024). There is a lack of robust evidence from RCTs to definitively inform the benefit-risk profile of TPT in people with diabetes and thus shape practice. In addition, in the absence of trial evidence, there are several factors that necessitates the careful consideration of the benefit and risk of TPT in this group.

First, the risk of TB in individuals with diabetes appears moderate (e.g., 1.5–3.5 fold)(Al-Rifai et al., 2017) compared to other high-risk groups (e.g. 10-fold increase in people living with HIV). Second, people with diabetes tend to be older and thus at higher risk for hepatotoxicity associated with TPT, particularly with the 6–9 months of daily isoniazid, which was until recently the only option for TPT. Third, there is a perceived burden on the health system; 366 million people in low- and middle-income countries where TB burden is high have diabetes, compared to only 30 million living with HIV. This would result in a vast number of individuals needing to be tested for TB infection and initiated on and followed up for TPT, potentially straining health systems. Further, isoniazid may adversely impact glycaemic control antagonizing the effects of sulphonylureas, and impairing insulin metabolism (Boadu et al., 2024a).

Two RCT are currently underway to evaluate the effectiveness of TPT in people with diabetes. The PROTID trial will assess the effectiveness and safety of a 3-month weekly rifapentine plus isoniazid regimen to prevent TB in people with diabetes in a placebo-controlled trial in Uganda and Tanzania (Ntinginya et al., 2022). The BALANCE trial is an open-label RCT evaluating the effectiveness and safety of a 1-month daily rifapentine plus isoniazid regimen compared to standard diabetes care in the Philippines and South Africa. Rifapentine-containing regimens are less hepatotoxic than daily isoniazid regimens and, together with their shorter durations, may alter the benefit-risk balance of TPT in favour of its use (Swindells et al., 2019).

An alternative, if not complementary, strategy to mitigate the risk of TB may be to target host factors. Studies suggest a significant association between poorly controlled diabetes and an increased risk of TB. For example, a cohort study reported that a 10 mg/dL increase in fasting plasma glucose was associated with a 6% increase in TB risk (Lee et al., 2016). Some immunological studies suggest that diabetes treatment restores impaired immune function, which may reverse the increased risk of TB. Metformin might also reduce the TB risk, independent of glycemic effects. A trial to clarify the TB preventive efficacy of metformin would be ideal but given that it is already the first-line treatment for people with diabetes, such a trial may not be feasible, unless tested in people without diabetes. Nevertheless, the ongoing RCT will help determine whether TPT offers additional benefits beyond standard diabetes treatment, including metformin use.

### Expert Opinion

The increasing prevalence of type 2 diabetes mellitus in areas of high tuberculosis incidence has created a syndemic. It is now well-recognized that diabetes predisposes to tuberculosis that is more severe, more difficult to treat and more likely to lead to complications. Quantitively more cases of tuberculosis associate with diabetes than with the best−recognized risk factor, HIV-1 co-infection. It is less well recognized that active tuberculosis associates with dysglycaemia which may progress to established diabetes. There is evidence that latent tuberculosis may also associate with impaired glucose tolerance. Underlying this bidirectional relationship are commonalities in immuno-metabolic dysregulation that may therefore represent important therapeutic opportunities. There is some evidence that drugs used to treat diabetes or its complications (e.g. metformin and statins) may have beneficial effects in resolving tuberculosis-induced immunopathology: these effects are not mediated via improved glycaemic control. Other compounds used in the treatment of diabetes such as calcium channel or angiotensin receptor blockers may also exert potential therapeutic benefit in tuberculosis. Conversely the use of corticosteroid therapy in some forms of tuberculosis will tend to exacerbate diabetes. There is a need to more precisely and quantitatively study the effects of tuberculosis and its treatment on glucose metabolism. Immunometabolic dysregulation can be investigated in controlled experimental laboratory models. Similarly, ongoing trials of tuberculosis prevention in people with diabetes should assess the effects of such therapy on glycaemic control and inflammatory markers. Randomized controlled trials of adjunctive therapies in tuberculosis have hitherto shown modest effects: standardized international case registries and experimental medicine studies of outcome in people with diabetes undergoing tuberculosis treatment with very accurate ascertainment, or experimental use, of concomitant medications may offer an alternative to conventional trials of adjunctive therapy which otherwise need to be large and therefore expensive to demonstrate clinically important results.

## Figures and Tables

**Table 1:** Summary of implications of TB and Diabetes comorbidity on treatment strategies for each condition

TB Treatment in Patients with Diabetes		
Implication:	Description:	Selected References:
Increased risk of TB	Increased susceptibility to latent *M.tb* infection, its progression to active disease, and *de novo* development of active TB.	(Shetty et al., 2024) (Kamper-Jorgensen et al., 2015; Koesoemadinata et al., 2017)
Delayed TB diagnosis	Longer time to confirmatory diagnosis and anti-TB treatment initiation.	(Chen et al., 2014; Wang et al., 2017)
Severe clinical disease	Disseminated and/or severely cavitating disease at diagnosis with greater clinical complexity if diabetes-related complications.	(Boadu et al., 2024b; Gil-Santana et al., 2016; Huang et al., 2017; Shetty et al., 2024; van Crevel and Critchley, 2021; Zhao et al., 2024)
Poor treatment outcomes	Increased likelihood of death during treatment, TB recurrence or relapse, and extended treatment duration.	(Kornfeld et al., 2023); (Wang et al., 2015); (Yanqiu et al., 2024)
Emergence of MDR-TB	Comparatively poor control of *M.tb* infection, increased mycobacterial proliferation, and considerably delayed sputum culture conversion to negative. Also, more frequent nosocomial acquisition of drug resistance.	(Zhao et al., 2024); (Abd El-Hamid El-Kady and Abdulrahman Turkistani, 2021; Rehman et al., 2023; van Crevel and Critchley, 2021)
Adverse drug reactions and events	Nephrotoxicity, hepatoxicity, visual acuity disturbances and hypothyroidism, among others, not uncommon due to polypharmacy and extended treatment duration.	(van Crevel and Critchley, 2021); (Muñoz-Torrico et al., 2017; Syed Suleiman et al., 2012; van Crevel and Critchley, 2021)
Post tuberculosis health status	Increased risk of long-term ill-health and disability from tissue destruction with adverse remodelling. Need for tailored treatment, including host directed adjuvants, as well as posttreatment follow up and rehabilitation.	(Restrepo, 2016)
**Diabetes Management in Patients with TB**		
Increased risk of diabetes	*M.tb* infection may be a novel risk factor for diabetes or a trigger for diabetes to manifest in individuals with predisposing factors.	(Magodoro et al., 2024; Pearson et al., 2019) Magee et al., 2022)
Diagnostic challenges	Difficulty classifying hyperglycaemia seen during active TB in patients of unknown diabetes status. Optimal timing of confirmatory re-testing to avoid misclassification of transient hyperglycaemia as diabetes remains to be established.	(Kubjane et al., 2020; Song et al., 2019)
Poor glycaemic control	Both TB and antituberculosis treatment can worsen blood glucose control in people with diabetes.	(Boadu et al., 2024a; Boadu et al., 2024b; Song et al., 2019)
Worsened glycaemic control	TB can worsen blood sugar control, making diabetes management more challenging.	(Boadu et al., 2024b)
Frequent monitoring and treatment titration	Blood glucose levels need to be monitored more frequently to adjust diabetes medications, including insulin requirements, among people with diabetes. Insulin requirements frequently increased, for example, due to stress and inflammation.	(Niazi and Kalra, 2012)
**Integrated Care Approach**		
Multidisciplinary care	Managing patients with both TB and diabetes often requires a team approach, including infectious disease specialists, endocrinologists, and primary care providers.	(Byashalira et al., 2023)
Patient education	Educating patients about the importance of adherence to both TB and diabetes treatment regimens is crucial for successful outcomes.	(Koesoemadinata et al., 2021)
Extended follow-up	Frequent follow-ups are necessary to monitor the progress of both conditions and make timely adjustments to treatment plans.	(Krishna and Jacob, 2000)
Nutritional support	Proper nutrition is crucial to support the immune system and manage both conditions effectively.	(Girishbhai Patel et al., 2024)

## Article highlights

- There is a syndemic of diabetes mellitus and *Mycobacterium tuberculosis* infection.
- Insulin resistance, hyperglycemia, pro-inflammatory cytokine release, aberrant NLRP3 inflammasome activation and oxidative stress occur in both TB and diabetes.
- The hypoglycemic and immunomodulatory effects of antidiabetic medications might deserve consideration as adjuvant host directed therapies in active TB.
- Opportunities for risk stratification and more precise adjuvant host directed therapies in active TB may exist.

## References

[R1] Abd El-Hamid El-Kady R, Abdulrahman Turkistani S (2021). The Footprint of Diabetes Mellitus on the Characteristics and Response to Anti-Tuberculous Therapy in Patients with Pulmonary Tuberculosis from Saudi Arabia. Infect Drug Resist.

[R2] Adewole OO, Omotoso BA, Ogunsina M, Aminu A, Ayoola O, Adedeji T, Awopeju OF, Sogaolu OM, Adewole TO, Odeyemi AO, Jiya E (2023). Atorvastatin improves sputum conversion and chest X-ray severity score. Int J Tuberc Lung Dis.

[R3] Aftab H, Ambreen A, Jamil M, Garred P, Petersen JH, Nielsen SD, Bygbjerg IC, Christensen DL (2017). High prevalence of diabetes and anthropometric heterogeneity among tuberculosis patients in Pakistan. Trop Med Int Health.

[R4] Agarwal P, Khan SR, Verma SC, Beg M, Singh K, Mitra K, Gaikwad AN, Akhtar MS, Krishnan MY (2014). Mycobacterium tuberculosis persistence in various adipose depots of infected mice and the effect of anti-tubercular therapy. Microbes Infect.

[R5] Agrawal NK, Kant S (2014). Targeting inflammation in diabetes: Newer therapeutic options. World J Diabetes.

[R6] Ahlstrom M, Lamberg-Allardt C (1999). Regulation of adenosine 3’,5’-cyclic monophosphate (cAMP) accumulation in UMR-106 osteoblast-like cells: role of cAMP-phosphodiesterase and cAMP efflux. Biochem Pharmacol.

[R7] Al-Rifai RH, Pearson F, Critchley JA, Abu-Raddad LJ (2017). Association between diabetes mellitus and active tuberculosis: A systematic review and meta-analysis. PLoS One.

[R8] Al-Tawfiq JA, Saadeh BM (2009). Radiographic manifestations of culture-positive pulmonary tuberculosis: cavitary or non-cavitary?. Int J Tuberc Lung Dis.

[R9] Alffenaar JC, Akkerman OW, van Hest R (2016). Statin Adjunctive Therapy for Tuberculosis Treatment. Antimicrob Agents Chemother.

[R10] Ambrosioni E, Bacchelli S, Esposti DD, Borghi C (2001). Anti-ischemic effects of angiotensin-converting enzyme inhibitors: a future therapeutic perspective. J Cardiovasc Pharmacol.

[R11] Amin SKM, Shabbier G, Wazir MN (2011). Frequency of pulmonary tuberculosis in patients with diabetes mellitus. Gomal Journal of Medical Sciences.

[R12] Arai M, Uchiba M, Komura H, Mizuochi Y, Harada N, Okajima K (2010). Metformin, an antidiabetic agent, suppresses the production of tumor necrosis factor and tissue factor by inhibiting early growth response factor-1 expression in human monocytes in vitro. J Pharmacol Exp Ther.

[R13] Arbues A, Brees D, Chibout SD, Fox T, Kammuller M, Portevin D (2020). TNF-alpha antagonists differentially induce TGF-beta1-dependent resuscitation of dormant-like Mycobacterium tuberculosis. PLoS Pathog.

[R14] Ayyappan JP, Vinnard C, Subbian S, Nagajyothi JF (2018). Effect of Mycobacterium tuberculosis infection on adipocyte physiology. Microbes Infect.

[R15] Benicky J, Sanchez-Lemus E, Pavel J, Saavedra JM (2009). Anti-inflammatory effects of angiotensin receptor blockers in the brain and the periphery. Cell Mol Neurobiol.

[R16] Bisht MK, Dahiya P, Ghosh S, Mukhopadhyay S (2023). The cause-effect relation of tuberculosis on incidence of diabetes mellitus. Front Cell Infect Microbiol.

[R17] Boadu AA, Yeboah-Manu M, Osei-Wusu S, Yeboah-Manu D (2024a). Tuberculosis and diabetes mellitus: The complexity of the comorbid interactions. International Journal of Infectious Diseases.

[R18] Boadu AA, Yeboah-Manu M, Osei-Wusu S, Yeboah-Manu D (2024b). Tuberculosis and diabetes mellitus: The complexity of the comorbid interactions. Int J Infect Dis.

[R19] Bohme J, Martinez N, Li S, Lee A, Marzuki M, Tizazu AM, Ackart D, Frenkel JH, Todd A, Lachmandas E, Lum J (2020). Metformin enhances anti-mycobacterial responses by educating CD8+ T-cell immunometabolic circuits. Nat Commun.

[R20] Brown JM, Ellis F (1969). The use of pyrimidine analogues in radiotherapy. Br J Radiol.

[R21] Bruiners N, Dutta NK, Guerrini V, Salamon H, Yamaguchi KD, Karakousis PC, Gennaro ML (2020). The anti-tubercular activity of simvastatin is mediated by cholesterol-driven autophagy via the AMPK-mTORC1-TFEB axis. J Lipid Res.

[R22] Brzostek A, Pawelczyk J, Rumijowska-Galewicz A, Dziadek B, Dziadek J (2009). Mycobacterium tuberculosis is able to accumulate and utilize cholesterol. J Bacteriol.

[R23] Byashalira KC, Chamba NG, Alkabab Y, Ntinginya NE, Affenaar JW, Heysell SK, Ramaiya KL, Lillebaek T, Bygbjerg IC, Christensen DL, Mpagama SG (2023). Point-of-care glycated hemoglobin a1c testing for the identification of hyperglycemia severity among individuals with dual tuberculosis and diabetes mellitus in Tanzania. Int J Mycobacteriol.

[R24] Cardona P, Cardona PJ (2019). Regulatory T Cells in Mycobacterium tuberculosis Infection. Front Immunol.

[R25] Carson P, Giles T, Higginbotham M, Hollenberg N, Kannel W, Siragy HM (2001). Angiotensin receptor blockers: evidence for preserving target organs. Clin Cardiol.

[R26] Chandra P, Grigsby SJ, Philips JA (2022). Immune evasion and provocation by Mycobacterium tuberculosis. Nat Rev Microbiol.

[R27] Chatterjee S, Yabaji SM, Rukhlenko OS, Bhattacharya B, Waligurski E, Vallavoju N, Ray S, Kholodenko BN, Brown LE, Beeler AB, Ivanov AR (2021). Channeling macrophage polarization by rocaglates increases macrophage resistance to Mycobacterium tuberculosis. iScience.

[R28] Chen HG, Liu M, Jiang SW, Gu FH, Huang SP, Gao TJ, Zhang ZG (2014). Impact of diabetes on diagnostic delay for pulmonary tuberculosis in Beijing. Int J Tuberc Lung Dis.

[R29] Chidambaram V, Ruelas Castillo J, Kumar A, Wei J, Wang S, Majella MG, Gupte A, Wang JY, Karakousis PC (2021). The association of atherosclerotic cardiovascular disease and statin use with inflammation and treatment outcomes in tuberculosis. Sci Rep.

[R30] Chung E, Jeong D, Mok J, Jeon D, Kang HY, Kim H, Kim H, Choi H, Kang YA (2024). Relationship between metformin use and mortality in tuberculosis patients with diabetes: a nationwide cohort study. Korean J Intern Med.

[R31] Cliff JM, Kaufmann SH, McShane H, van Helden P, O’Garra A (2015). The human immune response to tuberculosis and its treatment: a view from the blood. Immunol Rev.

[R32] Committee WGAbtGR (2014). WHO Guidelines for the Programmatic Management of Drug-Resistant Tuberculosis.

[R33] Cooper AM, Khader SA (2008). The role of cytokines in the initiation, expansion, and control of cellular immunity to tuberculosis. Immunol Rev.

[R34] Cross GB, Sari IP, Kityo C, Lu Q, Pokharkar Y, Moorakonda RB, Thi HN, Do Q, Dalay VB, Gutierrez E, Balanag VM (2023). Rosuvastatin adjunctive therapy for rifampicin-susceptible pulmonary tuberculosis: a phase 2b, randomised, open-label, multicentre trial. Lancet Infect Dis.

[R35] Crowley SD (2014). The cooperative roles of inflammation and oxidative stress in the pathogenesis of hypertension. Antioxid Redox Signal.

[R36] Das MK, Savidge B, Pearl JE, Yates T, Miles G, Pareek M, Haldar P, Cooper AM (2024). Altered hepatic metabolic landscape and insulin sensitivity in response to pulmonary tuberculosis. PLoS Pathog.

[R37] Dave JA, Lambert EV, Badri M, West S, Maartens G, Levitt NS (2011). Effect of nonnucleoside reverse transcriptase inhibitor-based antiretroviral therapy on dysglycemia and insulin sensitivity in South African HIV-infected patients. J Acquir Immune Defic Syndr.

[R38] Davuluri KS, Singh AK, Singh AV, Chaudhary P, Raman SK, Kushwaha S, Singh SV, Chauhan DS (2023). Atorvastatin Potentially Reduces Mycobacterial Severity through Its Action on Lipoarabinomannan and Drug Permeability in Granulomas. Microbiol Spectr.

[R39] Dhande I, Ma W, Hussain T (2015). Angiotensin AT2 receptor stimulation is anti-inflammatory in lipopolysaccharide-activated THP-1 macrophages via increased interleukin-10 production. Hypertens Res.

[R40] Duan H, Liu T, Zhang X, Yu A, Cao Y (2020). Statin use and risk of tuberculosis: a systemic review of observational studies. Int J Infect Dis.

[R41] Dutta NK, Bruiners N, Pinn ML, Zimmerman MD, Prideaux B, Dartois V, Gennaro ML, Karakousis PC (2016). Statin adjunctive therapy shortens the duration of TB treatment in mice. J Antimicrob Chemother.

[R42] Dutta NK, Bruiners N, Zimmerman MD, Tan S, Dartois V, Gennaro ML, Karakousis PC (2020). Adjunctive Host-Directed Therapy With Statins Improves Tuberculosis-Related Outcomes in Mice. J Infect Dis.

[R43] Eckold C, Kumar V, Weiner J, Alisjahbana B, Riza AL, Ronacher K, Coronel J, Kerry-Barnard S, Malherbe ST, Kleynhans L, Stanley K (2021). Impact of Intermediate Hyperglycemia and Diabetes on Immune Dysfunction in Tuberculosis. Clin Infect Dis.

[R44] English E, Idris I, Smith G, Dhatariya K, Kilpatrick ES, John WG (2015). The effect of anaemia and abnormalities of erythrocyte indices on HbA1c analysis: a systematic review. Diabetologia.

[R45] Esaki T, Roy K, Yao R, Galivan J, Sirotnak FM (1998). Cloning of mouse gamma-glutamyl hydrolase in the form of two cDNA variants with different 5’ ends and encoding alternate leader peptide sequences. Gene.

[R46] Fu CP, Lee CL, Li YH, Lin SY (2021). Metformin as a potential protective therapy against tuberculosis in patients with diabetes mellitus: A retrospective cohort study in a single teaching hospital. J Diabetes Investig.

[R47] Gallwitz B (2019). Clinical Use of DPP-4 Inhibitors. Front Endocrinol (Lausanne).

[R48] Gatfield J, Pieters J (2000). Essential role for cholesterol in entry of mycobacteria into macrophages. Science.

[R49] Gautam S, Shrestha N, Mahato S, Nguyen TPA, Mishra SR, Berg-Beckhoff G (2021). Diabetes among tuberculosis patients and its impact on tuberculosis treatment in South Asia: a systematic review and meta-analysis. Sci Rep.

[R50] Gil-Santana L, Almeida-Junior JL, Oliveira CA, Hickson LS, Daltro C, Castro S, Kornfeld H, Netto EM, Andrade BB (2016). Diabetes Is Associated with Worse Clinical Presentation in Tuberculosis Patients from Brazil: A Retrospective Cohort Study. PLoS One.

[R51] Girishbhai Patel D, Baral T, Jacob Kurian S, Malakapogu P, Saravu K, Sekhar Miraj S (2024). Nutritional status in patients with tuberculosis and diabetes mellitus: A comparative observational study. J Clin Tuberc Other Mycobact Dis.

[R52] Gonzalez-Muniz OE, Rodriguez-Carlos A, Santos-Mena A, Jacobo-Delgado YM, Gonzalez-Curiel I, Rivas-Santiago C, Navarro-Tovar G, Rivas-Santiago B (2024). Metformin modulates corticosteroids hormones in adrenals cells promoting Mycobacterium tuberculosis elimination in human macrophages. Tuberculosis (Edinb).

[R53] Guerra-De-Blas PDC, Bobadilla-Del-Valle M, Sada-Ovalle I, Estrada-Garcia I, Torres-Gonzalez P, Lopez-Saavedra A, Guzman-Beltran S, Ponce-de-Leon A, Sifuentes-Osornio J (2019). Simvastatin Enhances the Immune Response Against Mycobacterium tuberculosis. Front Microbiol.

[R54] Gurlek A, Kilickap M, Dincer I, Dandachi R, Tutkak H, Oral D (2001). Effect of losartan on circulating TNFalpha levels and left ventricular systolic performance in patients with heart failure. J Cardiovasc Risk.

[R55] Hagiwara S, Iwasaka H, Matumoto S, Hidaka S, Noguchi T (2009). Effects of an angiotensin-converting enzyme inhibitor on the inflammatory response in in vivo and in vitro models. Crit Care Med.

[R56] Heo E, Kim E, Jang EJ, Lee CH (2021). The cumulative dose-dependent effects of metformin on the development of tuberculosis in patients newly diagnosed with type 2 diabetes mellitus. BMC Pulm Med.

[R57] Huang CK, Huang JY, Chang CH, Tsai SJ, Shu CC, Wang HC, Chien KL (2024). The effect of statins on the risk of anti-tuberculosis drug-induced liver injury among patients with active tuberculosis: A cohort study. J Microbiol Immunol Infect.

[R58] Huang LK, Wang HH, Lai YC, Chang SC (2017). The impact of glycemic status on radiological manifestations of pulmonary tuberculosis in diabetic patients. PLoS One.

[R59] Huangfu P, Ugarte-Gil C, Golub J, Pearson F, Critchley J (2019). The effects of diabetes on tuberculosis treatment outcomes: an updated systematic review and meta-analysis. Int J Tuberc Lung Dis.

[R60] IDF (2021). International Diabetes Federation IDF Diabetes Atlas.

[R61] Istvan ES, Deisenhofer J (2001). Structural mechanism for statin inhibition of HMG-CoA reductase. Science.

[R62] Jeon CY, Murray MB (2008). Diabetes mellitus increases the risk of active tuberculosis: a systematic review of 13 observational studies. PLoS Med.

[R63] Jo YS, Han K, Kim D, Yoo JE, Kim Y, Yang B, Choi H, Sohn JW, Shin DW, Lee H (2021). Relationship between total cholesterol level and tuberculosis risk in a nationwide longitudinal cohort. Sci Rep.

[R64] Kamper-Jorgensen Z, Carstensen B, Norredam M, Bygbjerg IC, Andersen PH, Jorgensen ME (2015). Diabetes-related tuberculosis in Denmark: effect of ethnicity, diabetes duration and year of diagnosis. Int J Tuberc Lung Dis.

[R65] Kasal DA, Schiffrin EL (2012). Angiotensin II, Aldosterone, and Anti-Inflammatory Lymphocytes: Interplay and Therapeutic Opportunities. Int J Hypertens.

[R66] Khattak M, Rehman AU, Muqaddas T, Hussain R, Rasool MF, Saleem Z, Almalki MS, Alturkistani SA, Firash SZ, Alzahrani OM, Bahauddin AA (2024). Tuberculosis (TB) treatment challenges in TB-diabetes comorbid patients: a systematic review and meta-analysis. Ann Med.

[R67] Kim NH, Yu T, Lee DH (2014). The nonglycemic actions of dipeptidyl peptidase-4 inhibitors. Biomed Res Int.

[R68] Koesoemadinata RC, McAllister SM, Soetedjo NNM, Ratnaningsih Febni, Ruslami R, Kerry S, Verrall AJ, Apriani L, van Crevel R, Alisjahbana B, Hill PC (2017). Latent TB infection and pulmonary TB disease among patients with diabetes mellitus in Bandung, Indonesia. Trans R Soc Trop Med Hyg.

[R69] Koesoemadinata RC, McAllister SM, Soetedjo NNM, Santoso P, Ruslami R, Damayanti H, Rahmadika N, Alisjahbana B, van Crevel R, Hill PC (2021). Educational counselling of patients with combined TB and diabetes mellitus: a randomised trial. Public Health Action.

[R70] Kornfeld H, Procter-Gray E, Kumpatla S, Kane K, Li W, Magee MJ, Babu S, Viswanathan V (2023). Longitudinal trends in glycated hemoglobin during and after tuberculosis treatment. Diabetes Res Clin Pract.

[R71] Krishna S, Jacob JJ, Feingold KR, Anawalt B, Blackman MR, Boyce A, Chrousos G, Corpas E, de Herder WW, Dhatariya K, Dungan K, Hofland J, Kalra S (2000). Endotext.

[R72] Kubjane M, Berkowitz N, Goliath R, Levitt NS, Wilkinson RJ, Oni T (2020). Tuberculosis, Human Immunodeficiency Virus, and the Association With Transient Hyperglycemia in Periurban South Africa. Clin Infect Dis.

[R73] Kumar NP, Moideen K, Nancy A, Viswanathan V, Shruthi BS, Sivakumar S, Hissar S, Kornfeld H, Babu S (2019). Systemic RAGE ligands are upregulated in tuberculosis individuals with diabetes co-morbidity and modulated by anti-tuberculosis treatment and metformin therapy. BMC Infect Dis.

[R74] Kumar NP, Moideen K, Viswanathan V, Shruthi BS, Sivakumar S, Menon PA, Kornfeld H, Babu S (2018). Elevated levels of matrix metalloproteinases reflect severity and extent of disease in tuberculosis-diabetes co-morbidity and are predominantly reversed following standard anti-tuberculosis or metformin treatment. BMC Infect Dis.

[R75] Kurosaki E, Ogasawara H (2013). Ipragliflozin and other sodium-glucose cotransporter-2 (SGLT2) inhibitors in the treatment of type 2 diabetes: preclinical and clinical data. Pharmacol Ther.

[R76] Lee PH, Fu H, Lai TC, Chiang CY, Chan CC, Lin HH (2016). Glycemic Control and the Risk of Tuberculosis: A Cohort Study. PLoS Med.

[R77] Li WD, Li NP, Song DD, Rong JJ, Qian AM, Li XQ (2017). Metformin inhibits endothelial progenitor cell migration by decreasing matrix metalloproteinases, MMP-2 and MMP-9, via the AMPK/mTOR/autophagy pathway. Int J Mol Med.

[R78] Li X, Sheng L, Lou L (2020). Statin Use May Be Associated With Reduced Active Tuberculosis Infection: A Meta-Analysis of Observational Studies. Front Med (Lausanne).

[R79] Lin YH, Chen CP, Chen PY, Huang JC, Ho C, Weng HH, Tsai YH, Peng YS (2015). Screening for pulmonary tuberculosis in type 2 diabetes elderly: a cross-sectional study in a community hospital. BMC Public Health.

[R80] Lobato LS, Rosa PS, Ferreira Jda S, Neumann Ada S, da Silva MG, do Nascimento DC, Soares CT, Pedrini SC, Oliveira DS, Monteiro CP, Pereira GM (2014). Statins increase rifampin mycobactericidal effect. Antimicrob Agents Chemother.

[R81] Magee MJ, Khakharia A, Gandhi NR, Day CL, Kornfeld H, Rhee MK, Phillips LS (2022). Increased Risk of Incident Diabetes Among Individuals With Latent Tuberculosis Infection. Diabetes Care.

[R82] Magodoro I, Aluoch A, Claggett B, Nyirenda M, Siedner M, Wilkinson K, Wilkinson R, Ntusi N (2024). Insulin resistance, and not β-cell impairment, mediates association between Mycobacterium tuberculosis sensitization and type II diabetes mellitus among US adults. medRxiv.

[R83] Mahishale V, Avuthu S, Patil B, Lolly M, Eti A, Khan S (2017). Effect of Poor Glycemic Control in Newly Diagnosed Patients with Smear-Positive Pulmonary Tuberculosis and Type-2 Diabetes Mellitus. Iran J Med Sci.

[R84] Marx N, Federici M, Schutt K, Muller-Wieland D, Ajjan RA, Antunes MJ, Christodorescu RM, Crawford C, Di Angelantonio E, Eliasson B, Espinola-Klein C (2023). 2023 ESC Guidelines for the management of cardiovascular disease in patients with diabetes. Eur Heart J.

[R85] Matsumoto M, Einhaus D, Gold ES, Aderem A (2004). Simvastatin augments lipopolysaccharide-induced proinflammatory responses in macrophages by differential regulation of the c-Fos and c-Jun transcription factors. J Immunol.

[R86] Meng X, Zheng H, Wang X, Wang Y, Hu J, Zhao J, Gao Y (2024). Interaction of Glycemic Control and Statin Use on Diabetes-Tuberculosis Treatment Outcome: A Nested Case-Control Study. Can J Infect Dis Med Microbiol.

[R87] Meregildo-Rodriguez ED, Asmat-Rubio MG, Bardales-Zuta VH, Vasquez-Tirado GA (2024). Effect of calcium-channel blockers on the risk of active tuberculosis and mortality: systematic review and meta-analysis. Front Pharmacol.

[R88] Meregildo-Rodriguez ED, Asmat-Rubio MG, Zavaleta-Alaya P, Vasquez-Tirado GA (2022a). Effect of Oral Antidiabetic Drugs on Tuberculosis Risk and Treatment Outcomes: Systematic Review and Meta-Analysis. Trop Med Infect Dis.

[R89] Meregildo-Rodriguez ED, Chunga-Chevez EV, Gianmarco RL, Vasquez-Tirado GA (2022b). Further insights into to the role of statins against active tuberculosis: systematic review and meta-analysis. Infez Med.

[R90] Montero-Vega MT, Matilla J, Bazan E, Reimers D, De Andres-Martin A, Gonzalo-Gobernado R, Correa C, Urbano F, Gomez-Coronado D (2024). Fluvastatin Converts Human Macrophages into Foam Cells with Increased Inflammatory Response to Inactivated Mycobacterium tuberculosis H37Ra. Cells.

[R91] Muñoz-Torrico M, Caminero-Luna J, Migliori GB, D’Ambrosio L, Carrillo-Alduenda JL, Villareal-Velarde H, Torres-Cruz A, Flores-Vergara H, Martínez-Mendoza D, García-Sancho C, Centis R (2017). Diabetes is Associated with Severe Adverse Events in Multidrug-Resistant Tuberculosis. Arch Bronconeumol.

[R92] Munoz-Torrico M, Caminero Luna J, Migliori GB, D’Ambrosio L, Carrillo-Alduenda JL, Villareal-Velarde H, Torres-Cruz A, Flores-Ergara H, Martinez-Mendoza D, Garcia-Sancho C, Centis R (2017). Comparison of bacteriological conversion and treatment outcomes among MDR-TB patients with and without diabetes in Mexico: Preliminary data. Rev Port Pneumol (2006).

[R93] Naicker N, Rodel H, Perumal R, Ganga Y, Bernstein M, Benede N, Abdool Karim S, Padayacthi N, Sigal A, Naidoo K (2023). Metformin Increases Cell Viability and Regulates Pro-Inflammatory Response to Mtb. Infect Drug Resist.

[R94] Nasa JN, Brostrom R, Ram S, Kumar AM, Seremai J, Hauma M, Paul IA, Langidrik JR (2014). Screening adult tuberculosis patients for diabetes mellitus in Ebeye, Republic of the Marshall Islands. Public Health Action.

[R95] Nesto R (2004). C-reactive protein, its role in inflammation, Type 2 diabetes and cardiovascular disease, and the effects of insulin-sensitizing treatment with thiazolidinediones. Diabet Med.

[R96] Niazi AK, Kalra S (2012). Diabetes and tuberculosis: a review of the role of optimal glycemic control. J Diabetes Metab Disord.

[R97] Noubiap JJ, Nansseu JR, Nyaga UF, Nkeck JR, Endomba FT, Kaze AD, Agbor VN, Bigna JJ (2019). Global prevalence of diabetes in active tuberculosis: a systematic review and meta-analysis of data from 2.3 million patients with tuberculosis. Lancet Glob Health.

[R98] Ntinginya NE, Brake Te, Sabi I, Chamba N, Kilonzo K, Laizer S, Andia-Biraro I, Kibirige D, Kyazze AP, Ninsiima S, Critchley JA (2022). Rifapentine and isoniazid for prevention of tuberculosis in people with diabetes (PROTID): protocol for a randomised controlled trial. Trials.

[R99] Ottmani SE, Murray MB, Jeon CY, Baker MA, Kapur A, Lonnroth K, Harries AD (2010). Consultation meeting on tuberculosis and diabetes mellitus: meeting summary and recommendations. Int J Tuberc Lung Dis.

[R100] Padmapriydarsini C, Mamulwar M, Mohan A, Shanmugam P, Gomathy NS, Mane A, Singh UB, Pavankumar N, Kadam A, Kumar H, Suresh C (2022). Randomized Trial of Metformin With Anti-Tuberculosis Drugs for Early Sputum Conversion in Adults With Pulmonary Tuberculosis. Clin Infect Dis.

[R101] Pahari S, Kaur G, Negi S, Aqdas M, Das DK, Bashir H, Singh S, Nagare M, Khan J, Agrewala JN (2018). Reinforcing the Functionality of Mononuclear Phagocyte System to Control Tuberculosis. Front Immunol.

[R102] Pan SW, Feng JY, Yen YF, Chuang FY, Shen HS, Su VY, Chuang PH, Chan YJ, Su WJ (2020a). Metformin use and post-exposure incident tuberculosis: a nationwide tuberculosis-contact cohort study in Taiwan. ERJ Open Res.

[R103] Pan SW, Yen YF, Feng JY, Chuang PH, Su VY, Kou YR, Su WJ, Chan YJ (2020b). Opposite effects of statins on the risk of tuberculosis and herpes zoster in patients with diabetes: A population-based cohort study. Br J Clin Pharmacol.

[R104] Parihar SP, Guler R, Khutlang R, Lang DM, Hurdayal R, Mhlanga MM, Suzuki H, Marais AD, Brombacher F (2014). Statin therapy reduces the mycobacterium tuberculosis burden in human macrophages and in mice by enhancing autophagy and phagosome maturation. J Infect Dis.

[R105] Pearson F, Huangfu P, McNally R, Pearce M, Unwin N, Critchley JA (2019). Tuberculosis and diabetes: bidirectional association in a UK primary care data set. J Epidemiol Community Health.

[R106] Prasad-Reddy L, Isaacs D (2015). A clinical review of GLP-1 receptor agonists: efficacy and safety in diabetes and beyond. Drugs Context.

[R107] Ray KK, Seshasai SR, Erqou S, Sever P, Jukema JW, Ford I, Sattar N (2010). Statins and all-cause mortality in high-risk primary prevention: a meta-analysis of 11 randomized controlled trials involving 65,229 participants. Arch Intern Med.

[R108] Rehman AU, Khattak M, Mushtaq U, Latif M, Ahmad I, Rasool MF, Shakeel S, Hayat K, Hussain R, Alhazmi GA, Alshomrani AO (2023). The impact of diabetes mellitus on the emergence of multi-drug resistant tuberculosis and treatment failure in TB-diabetes comorbid patients: a systematic review and meta-analysis. Front Public Health.

[R109] Restrepo BI (2016). Metformin: Candidate host-directed therapy for tuberculosis in diabetes and non-diabetes patients. Tuberculosis (Edinb).

[R110] Roca FJ, Whitworth LJ, Prag HA, Murphy MP, Ramakrishnan L (2022). Tumor necrosis factor induces pathogenic mitochondrial ROS in tuberculosis through reverse electron transport. Science.

[R111] Rodriguez-Carlos A, Valdez-Miramontes C, Marin-Luevano P, Gonzalez-Curiel I, Enciso-Moreno JA, Rivas-Santiago B (2020). Metformin promotes Mycobacterium tuberculosis killing and increases the production of human beta-defensins in lung epithelial cells and macrophages. Microbes Infect.

[R112] Rottenberg ME, Huang L-K, Wang H-H, Lai Y-C, Chang S-C (2017). The impact of glycemic status on radiological manifestations of pulmonary tuberculosis in diabetic patients. Plos One.

[R113] Salindri AD, Gujabidze M, Kipiani M, Lomtadze N, Tukvadze N, Avaliani Z, Blumberg HM, Kornfeld H, Kempker RR, Magee MJ (2024). Metformin reduces the risk of poor treatment outcomes among individuals with rifampicin-resistant tuberculosis and type-2 diabetes mellitus. medRxiv.

[R114] Sathkumara HD, Hansen K, Miranda-Hernandez S, Govan B, Rush CM, Henning L, Ketheesan N, Kupz A (2020). Disparate Effects of Metformin on Mycobacterium tuberculosis Infection in Diabetic and Nondiabetic Mice. Antimicrob Agents Chemother.

[R115] Schmieder RE (2005). Mechanisms for the clinical benefits of angiotensin II receptor blockers. Am J Hypertens.

[R116] Schutz C, Davis AG, Sossen B, Lai RP, Ntsekhe M, Harley YX, Wilkinson RJ (2018). Corticosteroids as an adjunct to tuberculosis therapy. Expert Rev Respir Med.

[R117] Shakya N, Garg G, Agrawal B, Kumar R (2012). Chemotherapeutic interventions against tuberculosis. Pharmaceuticals (Basel).

[R118] Shetty S, Pappachan JM, Fernandez CJ (2024). Diabetes and tuberculosis: An emerging dual threat to healthcare. World J Diabetes.

[R119] Shi L, Jiang Q, Bushkin Y, Subbian S, Tyagi S (2019). Biphasic Dynamics of Macrophage Immunometabolism during Mycobacterium tuberculosis Infection. mBio.

[R120] Sierra C, de la Sierra A (2005). Antihypertensive, cardiovascular, and pleiotropic effects of angiotensin-receptor blockers. Curr Opin Nephrol Hypertens.

[R121] Silveira KD, Coelho FM, Vieira AT, Barroso LC, Queiroz-Junior CM, Costa VV, Sousa LF, Oliveira ML, Bader M, Silva TA, Santos RA (2013). Mechanisms of the anti-inflammatory actions of the angiotensin type 1 receptor antagonist losartan in experimental models of arthritis. Peptides.

[R122] Singhal A, Jie L, Kumar P, Hong GS, Leow MK, Paleja B, Tsenova L, Kurepina N, Chen J, Zolezzi F, Kreiswirth B (2014). Metformin as adjunct antituberculosis therapy. Sci Transl Med.

[R123] Song C, Xie W, Gong L, Ren M, Pan P, Luo B (2019). The relationship between HbA1c control levels and antituberculosis treatment effects: a meta-analysis. J Chin Med Assoc.

[R124] Stubbs B, Siddiqi K, Elsey H, Siddiqi N, Ma R, Romano E, Siddiqi S, Koyanagi A (2021). Tuberculosis and Non-Communicable Disease Multimorbidity: An Analysis of the World Health Survey in 48 Low- and Middle-Income Countries. Int J Environ Res Public Health.

[R125] Sun Q, Zhang Q, Xiao H, Cui H, Su B (2012). Significance of the frequency of CD4+CD25+CD127-T-cells in patients with pulmonary tuberculosis and diabetes mellitus. Respirology.

[R126] Swindells S, Ramchandani R, Gupta A, Benson CA, Leon-Cruz J, Mwelase N, Jean Juste MA, Lama JR, Valencia J, Omoz-Oarhe A, Supparatpinyo K (2019). One Month of Rifapentine plus Isoniazid to Prevent HIV-Related Tuberculosis. N Engl J Med.

[R127] Syed Suleiman SA, Ishaq Aweis DM, Mohamed AJ, Razakmuttalif A, Moussa MA (2012). Role of diabetes in the prognosis and therapeutic outcome of tuberculosis. Int J Endocrinol.

[R128] Tuite MF (1992). Antifungal drug development: the identification of new targets. Trends Biotechnol.

[R129] van Crevel R, Critchley JA (2021). The Interaction of Diabetes and Tuberculosis: Translating Research to Policy and Practice. Trop Med Infect Dis.

[R130] Vuorio A, Kovanen PT (2020). Statins as Adjuvant Therapy for COVID-19 to Calm the Stormy Immunothrombosis and Beyond. Front Pharmacol.

[R131] Wang CY, Liao KM, Wang YH, Chen KH, Chuang S, Liu CJ, Shu CC, Wang HC (2023). Dipeptidyl peptidase IV inhibitors and the risk of mycobacterial pulmonary infections in type 2 diabetes mellitus. J Infect Public Health.

[R132] Wang JY, Lee MC, Shu CC, Lee CH, Lee LN, Chao KM, Chang FY (2015). Optimal duration of anti-TB treatment in patients with diabetes: nine or six months?. Chest.

[R133] Wang Q, Ma A, Han X, Zhao S, Cai J, Kok FJ, Schouten EG (2017). Hyperglycemia is associated with increased risk of patient delay in pulmonary tuberculosis in rural areas. J Diabetes.

[R134] Wang Y, Zhou Y, Chen L, Cheng Y, Lai H, Lyu M, Zeng J, Zhang Y, Feng P, Ying B (2022). Metformin promotes smear conversion in tuberculosis-diabetes comorbidity and construction of prediction models. J Clin Lab Anal.

[R135] WHO (2024). WHO consolidated guidelines on tuberculosis: Module 6: Tuberculosis and comorbidities.

[R136] WHO (2025). Global tuberculosis report 2024.

[R137] Wu JY, Lee MG, Lee SH, Lee SH, Tsai YW, Hsu SC, Chang SS, Lee CC (2016). Angiotensin-Converting Enzyme Inhibitors and Active Tuberculosis: A Population-Based Study. Medicine (Baltimore).

[R138] Xiao W, Huang D, Li S, Zhou S, Wei X, Chen B, Zou G (2021). Delayed diagnosis of tuberculosis in patients with diabetes mellitus co-morbidity and its associated factors in Zhejiang Province, China. BMC Infect Dis.

[R139] Ye J, Zhu N, Sun R, Liao W, Fan S, Shi F, Lin H, Jiang S, Ying Y (2018). Metformin Inhibits Chemokine Expression Through the AMPK/NF-kappaB Signaling Pathway. J Interferon Cytokine Res.

[R140] Young F, Wotton CJ, Critchley JA, Unwin NC, Goldacre MJ (2012). Increased risk of tuberculosis disease in people with diabetes mellitus: record-linkage study in a UK population. J Epidemiol Community Health.

[R141] Yu X, Li L, Xia L, Feng X, Chen F, Cao S, Wei X (2019). Impact of metformin on the risk and treatment outcomes of tuberculosis in diabetics: a systematic review. BMC Infect Dis.

[R142] Zhang M, He JQ (2020). Impacts of metformin on tuberculosis incidence and clinical outcomes in patients with diabetes: a systematic review and meta-analysis. Eur J Clin Pharmacol.

[R143] Zhao L (2024). The Impact of Optimal Glycemic Control on Tuberculosis Treatment Outcomes in Patients With Diabetes Mellitus: Systematic Review and Meta-Analysis. JMIR public health and surveillance.

[R144] Zhao L, Gao F, Zheng C, Sun X (2024). The Impact of Optimal Glycemic Control on Tuberculosis Treatment Outcomes in Patients With Diabetes Mellitus: Systematic Review and Meta-Analysis. JMIR Public Health Surveill.

